# Alcohol and disease activity in axial spondyloarthritis: a cross-sectional study

**DOI:** 10.1007/s00296-018-3927-2

**Published:** 2018-01-10

**Authors:** Sizheng Zhao, Daniel Thong, Stephen J. Duffield, David Hughes, Nicola J. Goodson

**Affiliations:** 1 Department of Academic Rheumatology, Aintree University Hospital, Liverpool, UK; 2 Musculoskeletal Biology I, Institute of Ageing and Chronic Disease, University of Liverpool, Liverpool, UK; 3 Department of Biostatistics, Institute of Translational Medicine, University of Liverpool, Liverpool, UK

**Keywords:** Axial spondyloarthritis, Ankylosing spondylitis, Alcohol, Disease activity, Functional impairment, Quality of life

## Abstract

The objective of this study was to explore associations between alcohol consumption and disease activity in axial spondyloarthritis (axSpA). We conducted a cross-sectional study of axSpA participants meeting the ASAS criteria. Associations between self-reported current alcohol use and disease activity (BASDAI, spinal pain, ASDAS), functional impairment (BASFI), and quality of life were explored using multivariable linear models, adjusting for age, gender, symptom duration, use of TNF inhibition therapy, smoking, deprivation, and anxiety and depression (A&D). Within alcohol drinkers, effect of increased alcohol intake (defined as > 14 units/week) was explored with moderate drinking (≤ 14 units/week) as reference. The study cohort comprised 229 axSpA patients and 76% were male with mean age 46.5 years (SD ± 13.8). Alcohol drinking was reported by 64%, with a median of 6 units per week among drinkers. Compared with non-drinkers, drinkers had lower BASDAI (*β* = − 0.83; 95% CI − 1.49, − 0.17), ASDAS (*β* = − 0.36; 95% CI − 0.66, − 0.05) and BASFI (*β* = − 1.40; 95% CI − 2.12, − 0.68). These associations were in contrast to, and independent of, the detrimental effects of smoking, depression, and deprivation. Subgroup analysis in alcohol drinkers did not reveal significant associations between disease severity and increased alcohol intake. Stratified analyses by smoking revealed that in never-smokers without depression, alcohol was associated with greater reduction in disease activity: BASDAI (*β* = − 1.69; 95% CI − 2.93, − 0.45), ASDAS (*β* = − 0.60; 95% CI − 1.18, − 0.02). Favourable axSpA disease activity and function were observed in association with alcohol consumption in this cross-sectional study. Longitudinal study is required to explore whether this relationship is due to biological effects of alcohol on disease process or disease-associated behaviour modification.

## Introduction

Axial spondyloarthritis (axSpA) is a chronic inflammatory disease predominantly affecting the axial skeleton. Potentially modifiable environmental risk factors for increased disease activity and severity have received much research interest. There is emerging evidence that cigarette smoking is associated with a more severe disease phenotype in axSpA [1, 2]. Some studies have also suggested that dietary modification may reduce disease activity in AS [3]. However, the impact of alcohol consumption on axSpA disease activity and functional impairment has not been extensively studied, despite one study identifying an association between alcohol intake with radiographic damage in early disease [4].

Moderate alcohol intake has often been reported to beneficial for health [5] and is associated with reduced CRP in the general population [6]. In rheumatoid arthritis, alcohol consumption is inversely associated with risk [7], severity [8], extra-articular manifestations [9], and radiographic progression [10]. However, in psoriasis and psoriatic arthritis the converse in observed: alcohol consumption was associated with increased disease susceptibility independent of smoking [11, 12]. Therefore, the role of alcohol in inflammatory diseases, and particularly in axSpA, is far from clear. Lifestyle behaviours such as alcohol intake and smoking are frequently associated with demography and socioeconomic factors as well as psychological status. In any study examining impact of alcohol on inflammatory disease, adjustments for these potential confounding variables need to be considered. The aim of this study was to explore associations between alcohol consumption and disease activity and function in axSpA.

## Methods

Consecutive axSpA patients attending a tertiary referral spondyloarthritis service in Liverpool, UK, were recruited between 2010 and 2015. Patients were included if they fulfilled the ASAS criteria for axial SpA. Clinical data were collected at the initial clinical assessment including: age, gender, body mass index (BMI), smoking status, pack-years; meeting modified New York criteria for AS, symptom duration, duration since diagnosis, HLA-B27, extra-axial features; current use of NSAIDs, and tumour necrosis factor inhibition therapy (TNFi). Socioeconomic status was approximated using index of multiple deprivation (IMD) based on post-codes [13]. Decile 1 relates to the top 10% most deprived areas, whilst decile 10 represents the least deprived areas.

At the initial clinical assessment, participants reported frequency of drinking and estimated their average weekly alcohol consumption in units, with 1 unit is equal to 10 ml or 8 g of pure alcohol. They were categorised as drinkers or non-drinkers (including previous drinkers that have stopped for > 1 year). “Occasional” and “social” drinkers were classed as drinkers. Alcohol intake was used to divide drinkers into two groups, which, in this study, were labelled “moderate” (≤ 14 units/week) or “heavy” drinkers (> 14 units/week), for both genders as per current UK Department of Health guidelines [14]. This dichotomisation was performed, because retrospective patient estimates of units/week  were unlikely to be accurate and were often in multiples of 7.

Disease activity and functional status were assessed using numeric rating scale versions of the Bath AS Disease Activity Index (BASDAI), spinal pain, and Bath AS Functional Index (BASFI). AS disease activity score (ASDAS) was introduced after study commencement and was, therefore, missing at random. Quality of life was assessed by EuroQol (EQ5D-3L) using UK value sets and overall health status visual analogue scale (EQ-VAS). EQ5D index is anchored at 1 when there are no problems in all domains, and 0 as death [15]. Anxiety and depression (A&D) domain was extracted from EQ5D-3L and treated as a binomial variable recording the presence or absence of any self-reported A&D. Blood samples were taken on the day of assessment for ESR and CRP.

### Analysis

Statistical analyses were performed using Stata13. Comparison of patient and disease characteristics between drinking status and quantity were performed using Student’s *t* test for normally distributed, Mann–Whitney *U* test for non-normal, and Chi-squared or Fisher’s exact test for categorical variables.

Multivariable linear models were generated to explore the association between drinking status as the dichotomous independent variable and each measure of disease activity in turn. Models were adjusted for age, gender, symptom duration, use of TNFi, ever-smoking, IMD, and A&D. Additional models were generated to examine interactions between all covariates. Within drinkers, multivariable linear models with the above covariates were used to compare heavy drinkers to those reporting moderate intake. For sensitivity analysis, gender-specific tertiles of units/week were also generated within drinkers to explore intake quantity in multivariable models, using the lowest tertile as the reference group.

EQ5D index could not be adequately transformed and was, therefore, dichotomised by its median and analysed using logistic regression with the above covariates except A&D. AS diagnosis, use of NSAIDs, and HLA-B27 did not improve the model (assessed by likelihood ratio test) and were, therefore, not included. ESR and CRP were log-transformed prior to regression. Missing data were not imputed.

This study complies with the Declaration of Helsinki and was approved by the UK Research Ethics Committee (15/LO/1519).

## Results

The study recruited 238 participants with established axSpA of whom 229 (96%) had complete alcohol history. The cohort was predominantly male (76%) with mean age of 46.5 years (SD ± 13.8) and 83% met modified New York criteria for AS. The median symptom duration was 17.1 years [interquartile range (IQR) 8.4, 29.3]. HLA-B27 was measured in 141 (62%) and of these 60% were positive. TNFi treatment was used by 76 (33%). The median BASDAI was 5.7 (IQR 3.3, 7.6) and BASFI 5.7 (IQR 3.3, 7.6). ASDAS was available for 182 (79%) participants with mean of 2.7 (SD ± 1.1). Alcohol drinking was reported by 147 (64%). Amongst drinkers, the median quantity was 6 units per week (IQR 2, 20). Only two participants described themselves as ex-drinkers. There were no differences in participant or disease characteristics between those with alcohol history and the minority without (data not shown).

Participant and disease characteristics are shown in Table 1. Male participants were more likely to report drinking than females. No significant differences were seen between alcohol status and age, symptom duration, and proportion of AS, BMI, or extra-axial features, including psoriasis. There was no observed association between drinking and smoking. Most markers of disease severity were significantly worse in non-drinkers.

**Table 1:** Patient and disease characteristics compared between alcohol status and quantity categories

	No alcohol	Alcohol drinker	*P* value	Moderate drinking (≤ 14 units/week)	Heavy drinking (> 14 units/week)	*P* value
Number of patients	82 (36%)	147 (64%)		104 (71%)	43 (29%)	
Age	45.5 (14.3)	47.0 (13.6)	0.409	46.1 (14.0)	49.4 (12.4)	0.177
Male gender	**51 (62%)**	**122 (83%)**	**< 0.001**	**79 (76%)**	**43 (100%)**	**< 0.001**
BMI^a^	28.6 (4.7)	28.0 (5.7)	0.494	27.6 (5.8)	28.9 (5.3)	0.261
IMD decile	2 [1, 5]	2 [1, 6]	0.143	3 [1, 6]	2 [1, 5]	0.889
AS diagnosis	68 (83%)	122 (83%)	0.990	85 (82%)	37 (86%)	0.526
HLA-B27 status^a^	30 (53%)	55 (65%)	0.126	40 (65%)	15 (68%)	0.756
Symptom duration (years)	16.2 [6.5, 29.3]	17.2 [9.1, 29.6]	0.391	16.8 [8.4, 29.2]	20.4 [10.5, 32.5]	0.317
Diagnosis duration (years)	4.0 [0.7, 12.6]	5.2 [0.9, 15.2]	0.177	3.6 [0.8, 14.4]	8.0 [1.7, 17.6]	0.087
Ever-smoking	37 (45%)	70 (48%)	0.717	**42 (40%)**	**28 (65%)**	**0.006**
Pack-years	0 [0, 15]	0 [0, 20]	0.953	**0 [0, 10]**	**10 [0, 20]**	**0.008**
Self-reported A&D	43 (57%)	71 (52%)	0.505	46 (47%)	25 (64%)	0.070
BASDAI	**6.7 [3.7, 8.3]**	**5.3 [3, 7.2]**	**0.002**	5.3 [2.9, 7.2]	5.1 [3.1, 7.2]	0.914
Spinal pain	**7.0 [4.0, 9.0]**	**5.0 [3.0, 8.0]**	**0.008**	5 [3, 8]	6 [2, 8]	0.888
ASDAS^a^	**2.93 (1.22)**	**2.57 (1.09)**	**0.040**	2.49 (1.08)	2.78 (1.10)	0.199
BASFI	**6.5 [4.2, 8.4]**	**5.0 [2.1, 7.3]**	**0.001**	4.9 [2, 7.1]	5.7 [2.4, 7.5]	0.355
CRP (mg/L)^a^	3 [1, 10]	3 [1, 7]	0.582	3 [1, 7]	3 [1, 6]	0.434
ESR (mm/hr)^a^	9 [5, 23]	8 [5, 19]	0.412	8 [5, 20]	6.5 [2, 13.5]	0.113
EQ5D index^a^	0.52 [0.01, 0.69]	0.59 [0.09, 0.76]	0.068	**0.68 [0.16, 0.80]**	**0.16 [− 0.07, 0.76]**	**0.008**
EQ-VAS^a^	**53.1 (21.9)**	**43.7 (25.0)**	**0.008**	41.8 (2.83)	46.2 (3.30)	0.315
Peripheral joint involvement	20 (24%)	32 (23%)	0.751	23 (22%)	9 (23%)	0.924
Psoriasis	14 (17%)	23 (16%)	0.779	17 (16%)	6 (14%)	0.716
Uveitis	18 (22%)	45 (31%)	0.159	31 (30%)	14 (33%)	0.742
IBD	7 (9%)	14 (10%)	0.804	9 (9%)	5 (12%)	0.552
TNFi	27 (33%)	49 (33%)	0.950	**29 (28%)**	**20 (47%)**	**0.029**
NSAID	51 (62%)	106 (72%)	0.121	76 (73%)	30 (70%)	0.684

Statistically significant results are presented in bold

Data are presented in *n* (%), mean (SD), and median [IQR]

*A&D* anxiety and depression, *IMD* index of multiple deprivation, *ASDAS* Ankylosing Spondylitis Disease Activity Score, *EQ* EuroQoL, *IBD* inflammatory bowel disease, *TNFi* TNF inhibition therapy, *NSAID* non-steroidal anti-inflammatory drugs

^a^BMI complete data in 182; HLAB27 status known in 141; ASDAS in 182; CRP in 222; ESR in 221, EuroQol scores, and A&D in 213

Compared to moderate drinkers, heavy drinkers were more frequently male, were more likely to be smokers, and had greater pack-year smoking exposure. There were no differences in age and socioeconomic status. However, heavy drinkers reported significantly worse quality of life and in addition were more likely to be using TNFi therapy at the time of assessment. Alcohol status and quantity were not associated with IMD. However, ever-smoking (*P* = 0.01) and A&D (*P* = 0.01) were both associated with higher deprivation.

There was no association between alcohol drinking and A&D (*P* = 0.51), but there was a trend towards more A&D in heavy drinkers (64 vs 47%, *P* = 0.07). Ever-smokers more commonly reported A&D than never-smokers (54 vs 46%, *P* = 0.03). There was no association between any alcohol use and smoking (*P* = 0.72). However, heavy drinkers more frequently reported cigarette smoking than moderate drinkers (75 vs 40%, *P* = 0.01).

In multi-adjusted linear models without interaction terms, alcohol drinking was associated with lower disease activity (BASDAI *β* = − 0.83, 95% CI − 1.49, − 0.17; ASDAS *β* = − 0.36, 95% CI − 0.66, − 0.05) and functional impairment (BASFI *β* = − 1.40, 95% CI − 2.12, − 0.68) (Table 2). As covariates in this model, A&D was associated with higher disease activity assessed by all markers except CRP, whilst ever-smoking was associated with higher ASDAS, ESR, and CRP (Table 3). Deprivation (IMD) was associated with worse BASFI, quality of life, and ESR.

**Table 2:** Multivariable linear models comparing disease activity, functional impairment, and quality of life between alcohol drinkers and non-drinkers, adjusting for age, gender, symptom duration, use of TNFi, ever-smoking, IMD, and A&D

	*β* coefficient (95% CI) without interaction terms^a^	*β* coefficient (95% CI) in never-smokers^b^		*β* coefficient (95% CI) in ever-smokers if A&D is absent^b^	
		if A&D is present	if A&D is absent	if A&D is present	if A&D is absent
BASDAI	**− 0.83 (− 1.49, − 0.17)**	0.58 (**−** 0.66, 1.83)	**− 1.69 (− 2.93, − 0.45)**	0.32 (**−** 1.24, 1.88)	**− 1.64 (− 3.24, − 0.05)**
ASDAS	**− 0.36 (− 0.66, − 0.05)**	0.24 (**−** 0.36, 0.84)	**− 0.60 (− 1.18, − 0.02)**	0.35 (**−** 0.32, 1.02)	**− 0.71 (− 1.41, − 0.01)**
Spinal pain	**− 0.79 (− 1.56, − 0.02)**	0.44 (**−** 0.97, 1.84)	**− 2.11 (− 3.51, − 0.71)**	1.10 (**−** 0.79, 2.98)	**−** 0.44 (**−** 2.37, 1.49)
BASFI	**− 1.40 (− 2.12, − 0.68)**	**−** 0.18 (**−** 1.52, 1.15)	**− 2.25 (− 3.57, − 0.92)**	0.14 (**−** 1.56, 1.84)	**− 2.46 (− 4.20, − 0.73)**
EQ-VAS	**− 7.46 (− 13.6, − 1.34)**	11.4 (**−** 1.18, 24.0)	**− 13.9 (− 26.4, − 1.36)**	10.8 (**−** 2.98, 24.6)	**−** 7.84 (**−** 22.0, 6.33)
Ln(CRP)	**− 0.19 (− 0.56, 0.19)**	**−** 0.07 (**−** 0.78, 0.63)	**−** 0.13 (**−** 0.84, 0.57)	0.26 (**−** 0.67, 1.19)	**−** 0.55 (**−** 1.51, 0.42)
Ln(ESR)	**−** 0.15 (**−** 0.44, 0.15)	**−** 0.10 (**−** 0.61, 0.41)	**−** 0.06 (**−** 0.57, 0.45)	0.53 (**−** 0.22, 1.29)	**−** 0.11 (**−** 0.88, 0.67)

Statistically significant results are presented in bold

Total sample size for each regression model was 213 for BASDAI, BASFI and spinal pain, 181 for ASDAS, 206 EQ-VAS, 179 CRP, and 204 ESR

*BASDAI* Bath ankylosing spondylitis disease activity index, *ASDAS* Ankylosing Spondylitis Disease Activity Score, *BASFI* Bath AS functional index, *EQ-VAS* EuroQol visual analogue scale, *TNFi* TNF inhibitor, *IMD* index of multiple deprivation, *A&D* anxiety and depression

^a^Model: *y* = alcohol + age + gender + symptom duration + TNFi + eversmoking + IMD + A&D

^b^Model: *y* = alcohol + age + gender + symptom duration + TNFi + IMD + A&D + alcohol*A&D

**Table 3:** Effect sizes of anxiety and depression, ever-smoking, and index of deprivation as covariates in multivariable models of alcohol and disease activity, functional impairment, and quality of life

	Anxiety and depression	Ever-smoking	Index of multiple deprivation
BASDAI	**1.68 (1.01, 2.31)**	0.62 (− 0.02, 1.26)	− 0.07 (− 0.19, 0.04)
ASDAS	**0.75 (0.44, 1.06)**	**0.51 (0.21, 0.81)**	− 0.03 (− 0.08, 0.02)
Spinal pain	**1.70 (0.94, 2.45)**	0.47 (− 0.28, 1.22)	− 0.12 (− 0.26, 0.01)
BASFI	**1.78 (1.08, 2.48)**	0.51 (− 0.19, 1.20)	**− 0.14 (− 0.27, − 0.02)**
EQ-VAS	**19.9 (14.0, 25.8)**	2.22 (− 3.62, 8.07)	**− 1.83 (− 2.88, − 0.79)**
Ln(CRP)	0.30 (− 0.07, 0.66)	**0.45 (0.08, 0.81)**	0.05 (− 0.02, 0.11)
Ln(ESR)	**0.32 (0.03, 0.61)**	**0.37 (0.08, 0.65)**	**0.07 (0.02, 0.12)**

Statistically significant results are presented in bold

Total sample size for each regression model was 213 for BASDAI, BASFI, and spinal pain, 181 for ASDAS, 206 EQ-VAS, 179 CRP, and 204 ESR

*BASDAI* Bath ankylosing spondylitis disease activity index, *ASDAS* Ankylosing Spondylitis Disease Activity Score, *BASFI* Bath AS functional index, *EQ-VAS* EuroQol visual analogue scale

Alcohol status showed significant interactions with both ever-smoking and A&D in this model. Stratification by ever-smoking revealed an interaction between alcohol and A&D which was significant in ever-smokers but not in never-smokers. Within the never-smoking subgroup, a strong association between alcohol and reduced disease severity was observed (Table 2). Ever-smokers who consumed alcohol reported reduced BASDAI and BASFI, only in the absence of A&D.

Disease activity, functional impairment, and quality of life were not significantly different between heavy and moderate drinking. Sensitivity analyses using gender-specific tertiles also did not demonstrate an association between intake quantity and disease outcome measures. Multivariable logistic models of the dichotomised EQ5D index were not associated with alcohol status or quantity (data not shown).

## Discussion

Study of this cross-sectional axSpA cohort demonstrated an inverse association between alcohol drinking and disease activity and functional impairment. The association between alcohol and disease activity was in contrast to, and independent of, the detrimental effects of smoking, depression, and deprivation. A lower BASDAI of 0.8 to 1.6 is clinically relevant, as ≥ 2-unit improvement is considered a response to TNFi therapy [16]. In never-smokers, alcohol was associated with still greater reduction in disease activity. In ever-smokers, A&D appears to counteract the reduced disease activity seen with alcohol use. This is interesting, since alcohol and depression each increase the risk of the other and yet appear to have opposing effects on disease activity in axSpA patients.

Only two studies have explored alcohol consumption in axSpA. In a cross-sectional study, Zhang et al. focused on BASDAI (dichotomised at 4) and found increased odds of high BASDAI in moderate drinkers compared to non-drinkers, but analyses did not account for treatment or A&D and was not significant when adjusted for smoking [17]. Blachier et al. reported significantly increased odds of drinking in axSpA patients with radiographic changes at the sacroiliac joints and/or vertebrae [4]. The covariates were not explicit stated but did not include A&D, and no comments were made on disease activity.

In addition to more frequently reporting alcohol drinking, male patients were also more frequently categorised as heavy drinkers, which is consistent with population studies. Male gender in axSpA studies is usually associated with more severe disease. The finding that disease activity and functional impairment were both lower in alcohol drinkers, despite this male gender preponderance, is interesting.

Within the subgroup of participants reporting alcohol use, increasing tertiles of alcohol exposure was not associated with improved axSpA disease measures. This lack of dose–response effect is less suggestive of a biological protective effect from alcohol consumption. However, the relationship between alcohol intake and inflammation in population studies typically follows a U-shaped distribution with modest intake conferring protection which is lost at higher levels of alcohol consumption [18]. It is possible that the lack of association between axSpA disease measures with amount of alcohol consumed in this study could be due to the same phenomenon. Alcohol has been shown to suppress production of proinflammatory cytokines such as TNF and IL6 in human cell models, and moderate alcohol consumption is associated with lower levels of CRP, IL6, and soluble TNF receptors in healthy adults [19, 20]. Further study in larger disease population is required to explore this in more detail.

No associations between alcohol drinking and any extra-articular disease manifestations were seen in this study. This differs from the previous studies which have highlighted the association between alcohol use and disease susceptibility to psoriasis and psoriatic arthritis [11, 12].

There was no difference in self-reported A&D symptoms between drinkers and non-drinkers. This was not consistent with other studies where non-drinkers were less likely to develop A&D [21]. Self-reported A&D was significantly associated with worse disease activity and functional impairment with effect sizes larger than smoking or deprivation. There was no association between ever-smoking and drinking. However, heavy drinkers were more frequently smokers, which is consistent with general population studies showing a dose-dependent relationship between alcohol use and smoking [22]. Interestingly, ever-smoking was associated with only ASDAS, CRP, and ESR, but none of the other more subjective disease activity markers in multivariable models. Smoking’s previous reported effect on the Bath indices may be explained by confounding factors accounted for in this study. There was no difference in deprivation between alcohol status. However, increasing deprivation was independently associated with worse BASFI and quality of life. Despite the detrimental effects of smoking, depression, and deprivation, we observed lower disease activity and functional impairment in drinkers when these factors were adjusted for.

Studies of alcohol’s impact on health outcomes often report unexpected and conflicting results [23]. These inconsistencies may be explained by several methodological limitations. First, there are many confounders that should be considered. Alcohol drinking, depression, smoking, and socioeconomic status (SES) are interlinked and each have well-known effects on disease severity. The strength of this study is that these factors were adjusted for. However, this study cohort was recruited from an area with generally high levels of deprivation. Future studies should recruit a more mixed population. Second, many alcohol studies have been criticised for the heterogeneity of their non-drinker comparator, which often included occasional drinkers [23]. For this reason, two common responses—social and occasional drinking—were classified as drinkers in this study to provide a more homogenous comparator group. However, we were unable to perform sensitivity analyses of different categorisations. Third, it has been shown that alcohol intake is underestimated in surveys due to social desirability bias [23]. This study may be more susceptible to this bias given information was collected during clinical consultations. This may explain the low number of ex-drinkers. Like most alcohol studies, we focused on volume rather than frequency or quality. Future studies should distinguish patterns of drinking (such as binge-drinking) and also type of alcohol consumed (such as spirits, wines, beers). Wines are of particular interest as they are rich in anti-inflammatory polyphenols, such as resveratrol. These compounds inhibit TNF-induced nuclear factor kappa B (NF-κB) activation and may have protective properties in inflammatory arthritides [24].

The main limitation of this study was its cross-sectional design, which does not allow change in drinking behaviour to be considered. First, it is possible that those with more challenging disease activity may modify their behaviour and reduce alcohol intake to improve coping mechanisms. Second, studies have shown that measuring alcohol consumption during one time period in a participant’s life may be unreliable [6]. Repeated assessments of alcohol intake should be considered in future studies.

In conclusion, this study is the first to report that alcohol consumption is associated with reduced measures of axSpA disease severity than in non-drinkers. Further studies are needed to establish a causal link. These results cannot be used to endorse or promote the use of alcohol as both scientific evidence and guidelines are increasingly downplaying the previously held belief that moderate alcohol is beneficial. Furthermore, alcohol intake of three or more units per day is a recognised risk factor for osteoporosis [25], which is an important complication in axSpA.
